# Delayed Cervical Spinal Cord Injury After Thoracolumbar Scoliosis Correction: A Case Report and Review of the Literature

**DOI:** 10.7759/cureus.93146

**Published:** 2025-09-24

**Authors:** Wan Lye Cheong, Fadzrul Abbas Mohamed Ramlee, Teck Siang Lim, Mohd Afiq Muhamed Fuad, Mohd Hezery Harun

**Affiliations:** 1 Department of Orthopaedics, Faculty of Medicine and Health Sciences, Universiti Putra Malaysia, Serdang, MYS

**Keywords:** adolescent idiopathic scoliosis (ais), cervical spine injury, delayed neurologic deficit, posterior spinal fusion, scoliosis correction

## Abstract

We report the case of a 13-year-old female with progressive adolescent idiopathic scoliosis (AIS) who developed a delayed neurological deficit following T1-L3 posterior spinal fusion. Preoperatively, she had a 74° left proximal thoracic curve and a 96° right main thoracic curve. Surgery was uneventful, with stable intraoperative neuromonitoring and normal immediate neurological status. Twelve hours postoperatively, she developed progressive quadriplegia, preceded by episodes of hypotension. She was taken back to the theatre for implant removal and curve relaxation, but her deficit did not improve. Whole-spine MRI revealed extensive cervical cord oedema from C3 to T1. High-dose methylprednisolone and hemodynamic optimisation led to mild neurological improvement. After six months, she demonstrated partial recovery of upper limb strength, though she remained wheelchair-bound. At one year, she regained limited lower limb function and was able to ambulate short distances with a walking frame. This report highlights an extremely rare but catastrophic complication of scoliosis correction surgery and underscores the importance of vigilant postoperative monitoring.

## Introduction

Adolescent idiopathic scoliosis (AIS), the most common form of scoliosis, affects 1-3% of children aged 10-18 years [1]. It is characterised by a three-dimensional spinal deformity of unknown cause, presenting with lateral curvature and vertebral rotation. While many cases are mild and manageable with observation or bracing, curves exceeding 45-50° often require surgical correction to prevent progression, restore alignment, and improve quality of life [2]. The frequency of AIS surgeries has increased in recent decades, and although outcomes are generally favourable, complications occur in 5-23% of cases [2]. Early complications include neurological injury, dural tear, ophthalmologic or brachial plexus injuries, and positioning-related neuropathy, while late complications include infection, thromboembolism, pulmonary or gastrointestinal issues, and implant-related problems. Neurological injury, reported in 0.3-4% of cases, is among the most feared complications [2]. Intraoperative neuromonitoring using somatosensory evoked potentials (SSEP), transcranial motor evoked potentials (tcMEP), and the wake-up test enables early detection and intervention.

Despite these measures, delayed neurological deficits can occur in approximately 0.01% of cases, even when intraoperative neuromonitoring and immediate postoperative neurological examination were normal [3]. The cause remains unclear and is thought to relate to spinal cord ischaemia secondary to hypoperfusion or anaemia. These typically involve the level of spinal fusion, most commonly around T4. Cervical cord involvement is exceptionally rare. We present a case of delayed quadriplegia from cervical cord oedema following thoracolumbar scoliosis correction surgery.

## Case presentation

**Figure 1:**
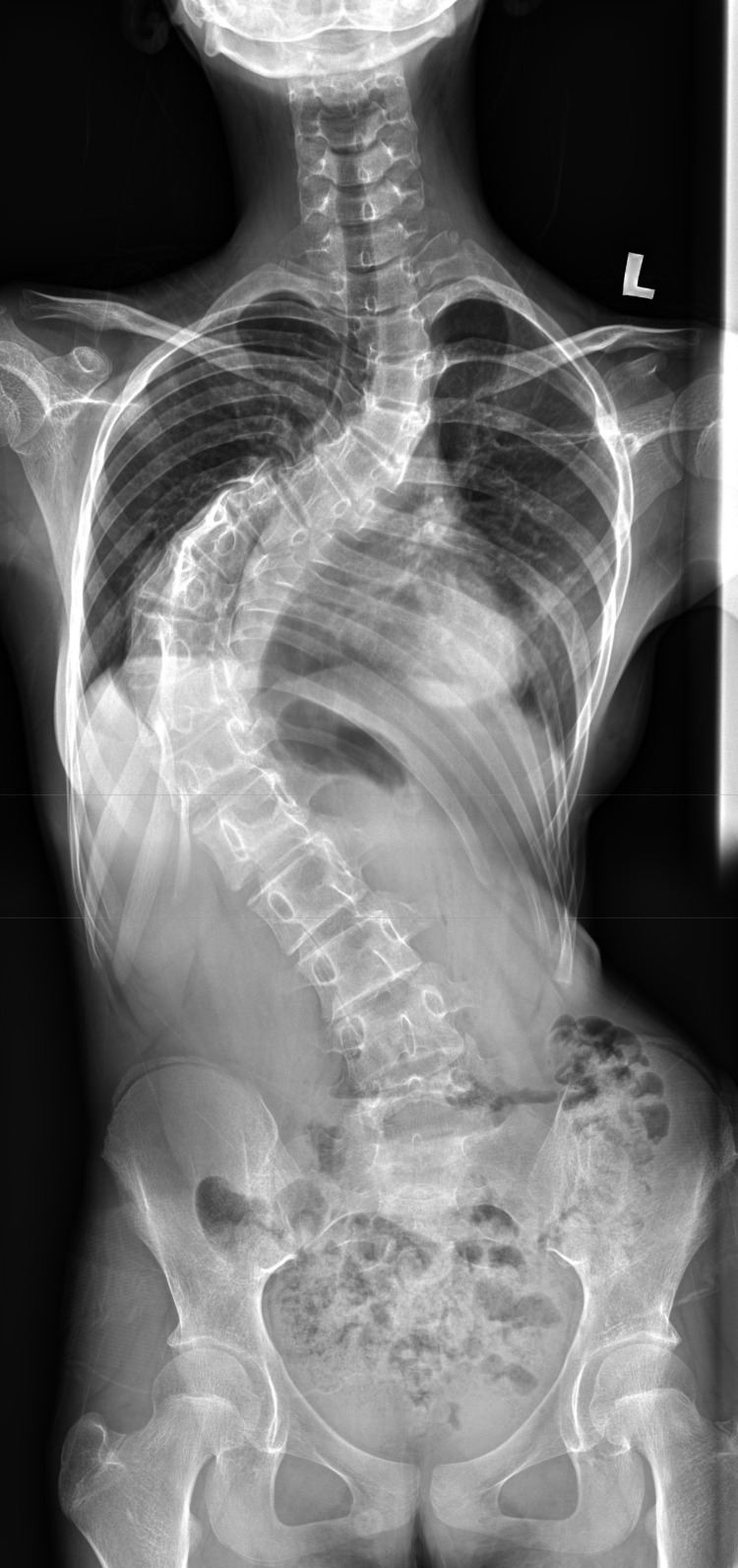
Whole spine radiograph (posteroanterior view) The image shows severe scoliosis with a right main thoracic curve and a left proximal thoracic curve

A 13-year-old pre-menarche female (50th percentile height) presented with progressive AIS and intermittent mechanical back pain, without neurological or respiratory symptoms. She was fully independent in daily activities. Birth and developmental history were unremarkable, and there was no family history of scoliosis or neuromuscular disease. Examination revealed shoulder and pelvic asymmetry with a right thoracic curve and a positive forward-bending test. No signs of spinal dysraphism or neurofibromatosis were present. Neurological assessment was normal. Radiographs demonstrated a 74° left proximal thoracic and a 96° right main thoracic curve (Figure 1). Preoperative MRI was normal, and a CT was obtained for surgical planning.

The patient underwent posterior spinal fusion and instrumentation from T1-L3. Under general anaesthesia, she was positioned prone on a Jackson table with neutral neck alignment. Pedicle screws were inserted, and the curve was corrected. Intraoperative neuromonitoring (SSEP and tcMEP) remained stable throughout. Blood loss was 1.1 L, and she received one unit of packed red cells. The estimated duration was six hours. Mean arterial pressure (MAP) was maintained above 70 mmHg. Pre- and postoperative haemoglobin levels were 13.4 g/dL and 10.7 g/dL, respectively. She was transferred intubated to the ICU for monitoring.

Two hours postoperatively, she developed transient hypotension (BP 84/60 mmHg, MAP 60-68 mmHg) with 350 mL drain output. IV tranexamic acid, fluids, and transfusion stabilised her vitals. Five hours later, another hypotensive episode (MAP 40 mmHg) necessitated temporary noradrenaline infusion and transfusion. Subsequently, MAP was maintained between 64 and 90 mmHg without inotropes.

**Figure 2:**
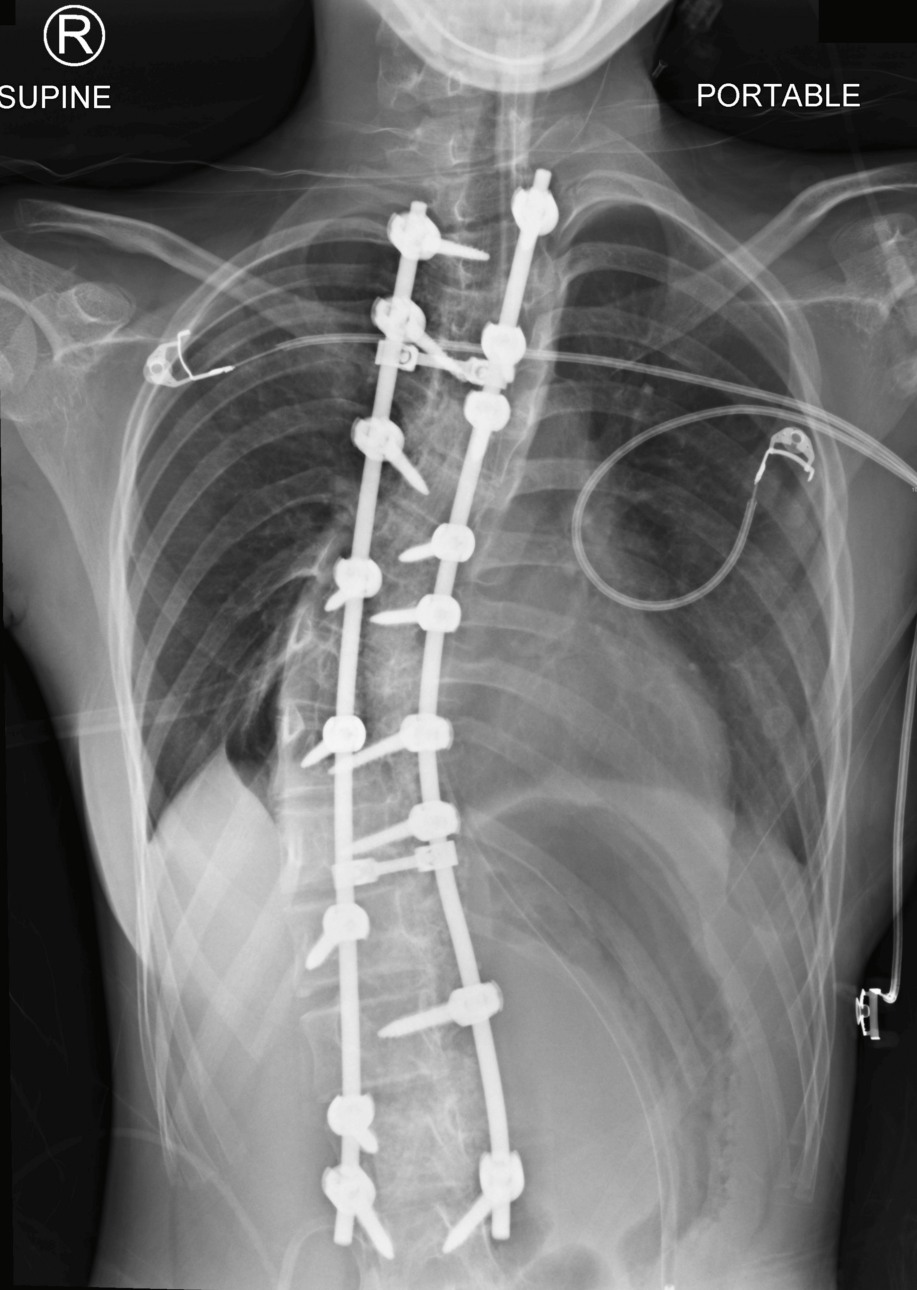
Postoperative radiograph (anteroposterior view) The image shows instrumentation and fusion of the thoracolumbar spine from T1 to L3 with modest curve correction

She was extubated six hours after surgery with stable hemodynamics and intact neurology. At 11 hours postoperatively, she developed quadriparesis (Medical Research Council (MRC) grade 4/5 elbow flexors, 2/5 wrist/elbow extensors, 1/5 finger flexors, 0/5 finger abductors; 0/5 bilateral lower limbs). Sensation was absent below T1. Haemoglobin was 10.7 g/dL. Radiographs showed no screw malposition (Figure 2). IV dexamethasone 8 mg was administered.

**Figure 3:**
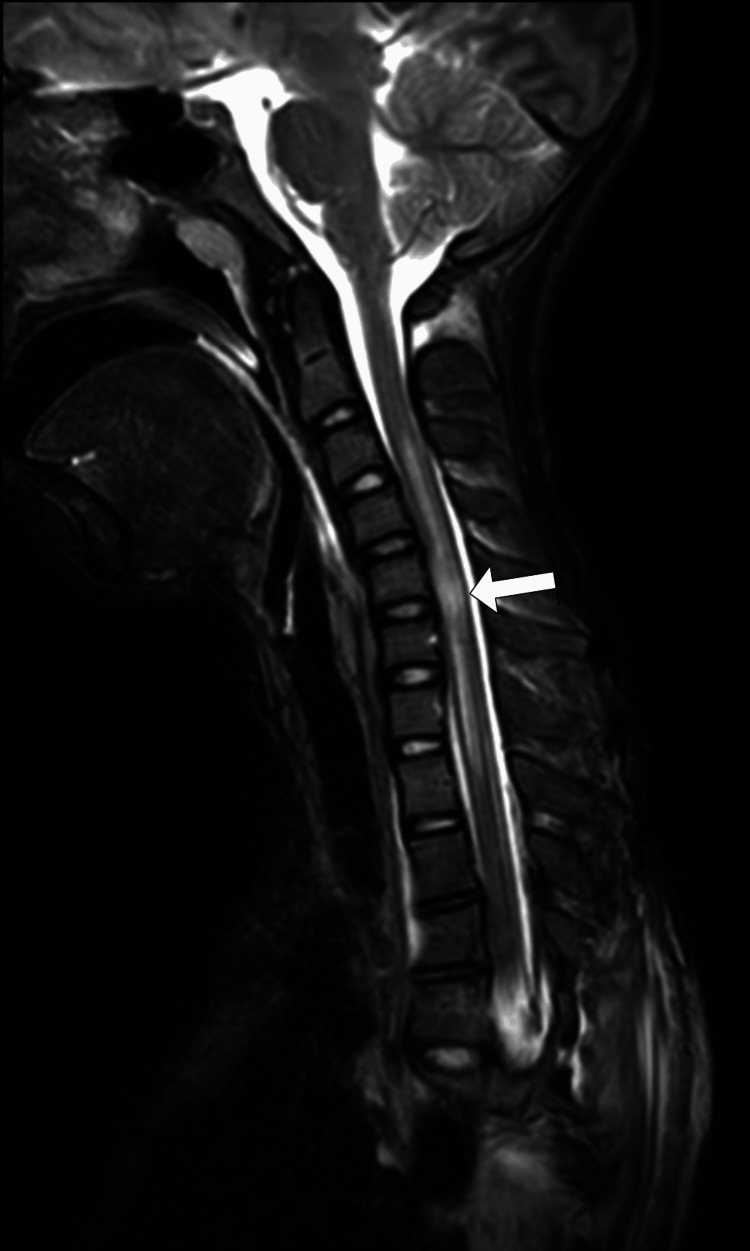
MRI cervical spine (T2W sagittal view) The image shows intramedullary hyperintense signal changes from C3 to T1, worst at C5 and C6 level (white arrow), suggestive of cord oedema MRI: magnetic resonance imaging

**Figure 4:**
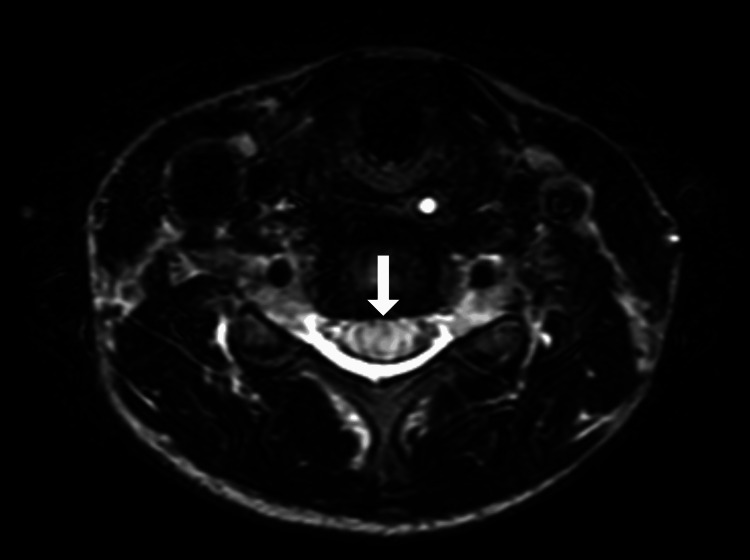
MRI cervical spine (T2W axial view) The image shows extensive intramedullary hyperintense signal changes at the level of C5/C6 (white arrow), suggestive of cord oedema MRI: magnetic resonance imaging

Urgent implant removal and curve relaxation were performed. Neuromonitoring did not show significant signal changes. Postoperatively, noradrenaline infusion was used to augment and maintain MAP at 80-90 mmHg. Neurological exam was unchanged. MRI revealed extensive cervical cord oedema from C3-T1, most severe at C5-C6 (Figures 3-4). No thoracic/lumbar cord compression or hematoma was seen. High-dose methylprednisolone was given per the acute spinal cord injury protocol.

The patient remained neurologically impaired after 48 hours. Prolonged ventilation was required, complicated by nosocomial infections. Rehabilitation included respiratory support, neurogenic bladder/bowel care, pressure sore prevention, and psychological support. Ventilation was weaned off after three months. At six months, upper limb strength improved (MRC 4-5/5 elbow/wrist extensors, 3/5 finger abductors), but she remained paraplegic. Sensation partially recovered in the right lower limb. At one year, she achieved MRC 2-3/5 power in lower limbs and ambulated short distances with a walking frame.

## Discussion

Neurological deficits after scoliosis correction are devastating. While intraoperative neuromonitoring reduces intraoperative risks, delayed neurological deficits, occurring hours to days after surgery, remain perplexing, particularly when the immediate postoperative neurological status is normal. The aetiology is multifactorial. Deficits may result from mechanical compression (e.g., hematoma, malpositioned screw, vertebral translation) or ischaemia [3]. Prompt decompression of compressive lesions is associated with better outcomes, whereas ischaemic causes often carry a poorer prognosis [3,4]. Perioperative hypotension, anaemia, excessive distraction, and altered vascular supply contribute to vascular compromise in already vulnerable spinal cords. Postoperative vasospasm and cord oedema may further impair perfusion. Animal studies have demonstrated that distraction can lead to microvessel alteration, hypoxia, and mitochondrial dysfunction, resulting in secondary injury [5]. This explains the delayed onset of neurological symptoms.

Most deficits occur at the site of distraction. In sheep models, blood flow reduction and tissue injury were greatest at the distraction level [6]. Human thoracic cord vascular anatomy, with relatively sparse and segmental supply, renders this region particularly susceptible to circulatory disturbances [7]. By contrast, ischaemic injury at a distant level, as in our case with cervical cord oedema, is exceptionally rare. Only a limited number of reports describe cervical cord involvement following thoracolumbar scoliosis correction [8-14]. Reported possible risk factors include female sex, perioperative hypoperfusion, and osteotomies [8]. Up to 85% of delayed deficits occur within 48 hours, with over half in the first 24 hours [4]. This highlights the importance of vigilant monitoring in the immediate postoperative period. Subtle weakness, sensory changes, or unexplained hemodynamic instability should prompt urgent evaluation. Close attention to MAP, haemoglobin, oxygenation, and fluid status is crucial. Sedatives and opioids may mask early neurological changes and exacerbate hypoventilation-related hypoperfusion [10].

Once a delayed deficit occurs, management can be challenging. Imaging may help exclude compressive pathology, although metallic instrumentation can limit accuracy. In cases of suspected ischaemia, strategies include urgent implant removal or decompression, strict MAP augmentation, transfusion to correct anaemia, and minimisation of sedatives [3,6]. In patients at risk of delayed neurologic injury, particularly those with intraoperative neuromonitoring abnormalities, Auerbach et al. proposed maintaining MAP >65 mmHg intraoperatively, and 80-90 mmHg for at least 48 hours following a deficit, together with transfusion to maintain physiologic haemoglobin, ICU-level monitoring, and the use of titanium implants to improve postoperative imaging sensitivity [3].

Despite these recommendations, several variables remain contentious. Optimal MAP targets are undefined. While hypotension is a recognised risk, not all patients with intraoperative MAP <60 mmHg develop deficits [15]. Schwartz et al. observed that alerts were triggered when MAP dropped below 59 mmHg, leading them to target MAP 80-90 mmHg postoperatively, with fluid resuscitation proving more effective than vasopressors [16]. Fletcher et al. similarly advised 75-85 mmHg during instrumentation, and >85 mmHg for complex procedures, with adjustments for children to avoid intracranial haemorrhage [17].

Patients experiencing hypotension within the first four hours postoperatively are more likely to have recurrent events and are at increased risk of neurological deficit [18]. Elevating MAP may mitigate this risk. However, the optimal duration is unknown, with authors reporting durations of 48 hours to seven days [9]. For intubated or sedated patients with unreliable neurological examination, prolonged MAP augmentation of at least 48-72 hours is often recommended [17]. The role of haemoglobin is also debated. Preoperative anaemia is linked to increased complications, although not specifically to neurological injury [19]. Given that haemoglobin may fall by 4 g/dL within two days postoperatively, some authors recommend a preoperative value at least 5 g/dL above the transfusion threshold (7-8 g/dL) [20].

Outcomes are variable: approximately 50% of patients achieve full recovery, 32% partial recovery, and 18% no recovery [4]. The prognosis of cervical cord injury following thoracolumbar instrumentation remains uncertain due to its extreme rarity. Nevertheless, awareness of this potential complication is vital, and it should be clearly communicated to the surgical team, anaesthesiologists, nursing staff, patients, and their families.

## Conclusions

Delayed cervical spinal cord injury following thoracolumbar scoliosis correction is an exceptionally rare but catastrophic complication. Despite stable intraoperative monitoring, postoperative hypotension may precipitate ischaemic cord injury at distant levels. Vigilant postoperative monitoring, early recognition of subtle deficits, and aggressive hemodynamic support are critical. Prognosis is guarded, but recovery is possible with multidisciplinary care and long-term rehabilitation.

## References

[1] Dunn J, Henrikson NB, Morrison CC, Blasi PR, Nguyen M, Lin JS (2018). Screening for adolescent idiopathic scoliosis: evidence report and systematic review for the US Preventive Services Task Force. JAMA.

[2] Murphy RF, Mooney JF 3rd (2016). Complications following spine fusion for adolescent idiopathic scoliosis. Curr Rev Musculoskelet Med.

[3] Auerbach JD, Kean K, Milby AH (2016). Delayed postoperative neurologic deficits in spinal deformity surgery. Spine (Phila Pa 1976).

[4] Lv H, Zhang Z, Yang A (2024). Delayed postoperative neurological deficits from scoliosis correction: a case series and systematic review on clinical characteristics, treatment, prognosis, and recovery. Eur Spine J.

[5] Wu D, Zheng C, Wu J, Xue J, Huang R, Wu D, Song Y (2017). The pathologic mechanisms underlying lumbar distraction spinal cord injury in rabbits. Spine J.

[6] Busch DR, Lin W, Cai C (2020). Multi-site optical monitoring of spinal cord ischemia during spine distraction. J Neurotrauma.

[7] Zhang Z, Nonaka H, Hatori T (1997). The microvasculature of the spinal cord in the human adult. Neuropathology.

[8] Lovi A, Manfroni F, Luca A, Babbi L, Brayda-Bruno M (2022). Delayed postoperative cervical spinal cord ischemic lesion after a thoracolumbar fusion for syndromic scoliosis: a case report and systematic review of the literature. Childs Nerv Syst.

[9] Kristobak A, Helgeson MD, Jex J (2019). Cervical cord injury following posterior spinal fusion in a patient with adolescent idiopathic scoliosis: a case report. JBJS Case Connect.

[10] Samtani RG, Bernatz JT, Halanski MA, Noonan KJ (2019). Cervical spine injury following thoracic spinal fusion for adolescent idiopathic scoliosis. Cureus.

[11] Youlo ST, Merrick MT, Cassidy JA, Halanski MA (2013). Cervical spinal cord injury after thoracic spinal instrumentation: a case series. Spine (Phila Pa 1976).

[12] Dapunt UA, Mok JM, Sharkey MS, Davis AA, Foster-Barber A, Diab M (2009). Delayed presentation of tetraparesis following posterior thoracolumbar spinal fusion and instrumentation for adolescent idiopathic scoliosis. Spine (Phila Pa 1976).

[13] Papadopoulos E, Falloon M, Mardjetko S, Boachie-Adjei O (2023). Delayed-postoperative quadriparesis following posterior thoracolumbar fusion for adolescent idiopathic scoliosis: a case series. Brain Spine.

[14] Alam M, Shufflebarger HL, Rush AJ, Rosas S, Lavelle WF, Sponseller PD, Asghar J (2020). Delayed quadriparesis after posterior spinal fusion for scoliosis: a case series. Spine Deform.

[15] Hewson DW, Bedforth NM, Hardman JG (2018). Spinal cord injury arising in anaesthesia practice. Anaesthesia.

[16] Schwartz DM, Auerbach JD, Dormans JP (2007). Neurophysiological detection of impending spinal cord injury during scoliosis surgery. J Bone Joint Surg Am.

[17] Fletcher ND, Ghag R, Hedequist DJ (2023). Perioperative blood pressure management for patients undergoing spinal fusion for pediatric spinal deformity. J Pediatr Soc North Am.

[18] Haber LL, Womack ED, Sathyamoorthy M, Moss JA, Shrader MW (2018). Who needs a pediatric intensive care unit after posterior spinal fusion for adolescent idiopathic scoliosis?. Spine Deform.

[19] De la Garza Ramos R, Goodwin CR, Abu-Bonsrah N (2016). Patient and operative factors associated with complications following adolescent idiopathic scoliosis surgery: an analysis of 36,335 patients from the Nationwide Inpatient Sample. J Neurosurg Pediatr.

[20] van Popta D, Stephenson J, Patel D, Verma R (2014). The pattern of blood loss in adolescent idiopathic scoliosis. Spine J.

